# Natural Killer T (NKT) Cells and Periodontitis: Potential Regulatory Role of NKT10 Cells

**DOI:** 10.1155/2021/5573937

**Published:** 2021-09-20

**Authors:** Samanta Melgar-Rodríguez, Emilio A. Cafferata, Nicolás I. Díaz, Miguel A. Peña, Luis González-Osuna, Carolina Rojas, Alfredo Sierra-Cristancho, Angélica M. Cárdenas, Jaime Díaz-Zúñiga, Rolando Vernal

**Affiliations:** ^1^Periodontal Biology Laboratory, Faculty of Dentistry, Universidad de Chile, Santiago, Chile; ^2^Department of Conservative Dentistry, Faculty of Dentistry, Universidad de Chile, Santiago, Chile; ^3^Department of Periodontology, School of Dentistry, Universidad Científica del Sur, Lima, Peru; ^4^Faculty of Dentistry, Universidad Andres Bello, Santiago, Chile; ^5^Health Sciences Division, Faculty of Dentistry, Universidad Santo Tomás, Bucaramanga, Colombia

## Abstract

Natural killer T (NKT) cells constitute a unique subset of T lymphocytes characterized by specifically interacting with antigenic glycolipids conjugated to the CD1d receptor on antigen-presenting cells. Functionally, NKT cells are capable of performing either effector or suppressor immune responses, depending on their production of proinflammatory or anti-inflammatory cytokines, respectively. Effector NKT cells are subdivided into three subsets, termed NKT1, NKT2, and NKT17, based on the cytokines they produce and their similarity to the cytokine profile produced by Th1, Th2, and Th17 lymphocytes, respectively. Recently, a new subgroup of NKT cells termed NKT10 has been described, which cooperates and interacts with other immune cells to promote immunoregulatory responses. Although the tissue-specific functions of NKT cells have not been fully elucidated, their activity has been associated with the pathogenesis of different inflammatory diseases with immunopathogenic similarities to periodontitis, including osteolytic pathologies such as rheumatoid arthritis and osteoporosis. In the present review, we revise and discuss the pathogenic characteristics of NKT cells in these diseases and their role in the pathogenesis of periodontitis; particularly, we analyze the potential regulatory role of the IL-10-producing NKT10 cells.

## 1. Introduction

The first evidence of the existence of Natural Killer T (NKT) cells appeared in 1987 [1–3]. Specifically, when a particular population of *αβ*T lymphocytes, expressing the V*β*8 chain of the T-cell receptor (TCR) more frequently than in conventional T lymphocytes and lacking the expression of CD4 and CD8 markers, was described [1–3]. However, it was not until 1995 that NKT cells were formally described as a subset of T lymphocytes with characteristics similar to Natural Killer (NK) cells, characterized by the expression of the NK marker NK1.1 [4–6]. From these studies, the interest in NKT cells has gradually increased due to their possible role in the pathogenesis of several inflammatory and autoimmune diseases, in particular when a subpopulation of NKT cells with promising immunoregulatory functions was reported [2, 3, 7].

**Figure 1:**
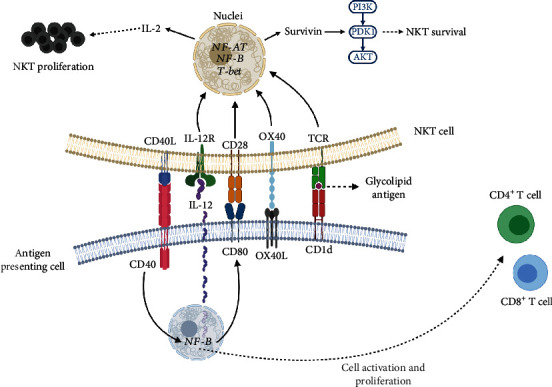
Antigenic presentation and activation of NKT cells. During antigen presentation, NKT cells recognize, via their TCR, the glycolipid antigen conjugated to the CD1d receptor expressed by antigen-presenting cells. In this context, the costimulatory ligand/receptor interactions between OX40L/OX40, CD80/CD28, and IL-12/IL-12R promote the activation of the transcription factors NF-AT, NF-*κ*B, and T-bet. In turn, these activations lead to the production of IL-2 and survivin, which promote NKT cell proliferation and survival by activation of the PI3K-PDK1-AKT signaling pathway. Furthermore, the CD40/CD40L interaction promotes the activation of NF-*κ*B in antigen-presenting cells, which induces IL-12 and CD80 production and promotes the CD4^+^ and CD8^+^ T lymphocyte activation and proliferation. AKT, protein kinase B; CD, cluster of differentiation; IL, interleukin; NF-AT; nuclear factor of activated T cells; NF-*κ*B, nuclear factor *κ*B; NKT, Natural Killer T cell; OX40, tumor necrosis factor receptor superfamily member 4; OX40L, tumor necrosis factor receptor superfamily member 4 ligand; PDK1, pyruvate dehydrogenase lipoamide kinase isozyme 1; PI3K, phosphatidylinositol-3-kinase; T-bet, transcription factor T-box; TCR, T-cell receptor. (Created with http://biorender.com).

NKT cells were preliminarily characterized by expressing an invariant chain of the TCR: in humans, the V*α*24/J*α*18 associated with the V*β*11 chain, and in mice, the V*α*14/J*α*18 chain associated with the V*β*2, V*β*8, or V*β*7 chain, together with the memory T lymphocyte marker CD45RO [8, 9]. Overall, NKT cells have demonstrated an important capacity to produce a diverse milieu of proinflammatory and anti-inflammatory cytokines, as well as costimulatory molecules, to promote cell activation [8, 9]. In relation to antigen recognition, NKT cells are specifically sensitive to glycolipid antigens conjugated to a specific receptor termed CD1d, a class Ib monomorphic molecule expressed by antigen-presenting cells (APCs), independent from the major histocompatibility complex (MHC) molecules [5, 10, 11]. Thus, CD1d, characterized by the expression of three extracellular domains (*α*1, *α*2, and *α*3) and formation of a heterodimer with *β*2-microglobulin, acts as a non-classical MHC I molecule [2, 11]. Indeed, the CD1d/*β*2-microglobulin complex conjugates with glycolipid antigens that, together with costimulatory molecules, promote the activation, proliferation, and selective differentiation of NKT cells [12] (Figure 1).

The NKT cell number and function are disturbed in a variety of autoimmune and inflammatory diseases; however, it still remains unclear whether these changes are directly related to the etiopathogenesis of these diseases [13]. Certainly, NKT cell functional deficiency has been proved to increase the severity in multiple mouse models for autoimmunity; however, in some models, NKT cell deficiency had no effect or even ameliorated disease, suggesting a complex relationship between autoimmunity and NKT cells [13, 14]. In humans, IFN-*γ* and IL-17-producing NKT cells have been identified in affected tissues in some inflammatory conditions [15–17], suggesting that the pathogenic role of NKT cells varies depending on the functional NKT subset involved.

## 2. Maturation and Differentiation of NKT Cells

In mice, NKT cells can be divided into two main groups, type I and type II NKT cells [11, 18]. Type I NKT cells express an invariant TCR *α*-chain associated with a TCR *β*-chain, the reason they are also called invariant NKT (iNKT) cells [11]. Otherwise, type II NKT cells express a diverse repertoire of TCRs similar to conventional T lymphocytes, the reason they are called diverse NKT (dNKT) cells [11, 13]. Both types of NKT cells recognize glycolipid antigens conjugated to CD1d; however, after activation, iNKT cells can display either proinflammatory or anti-inflammatory activities, while dNKT cells perform mainly anti-inflammatory functions [13, 18].

**Figure 2:**
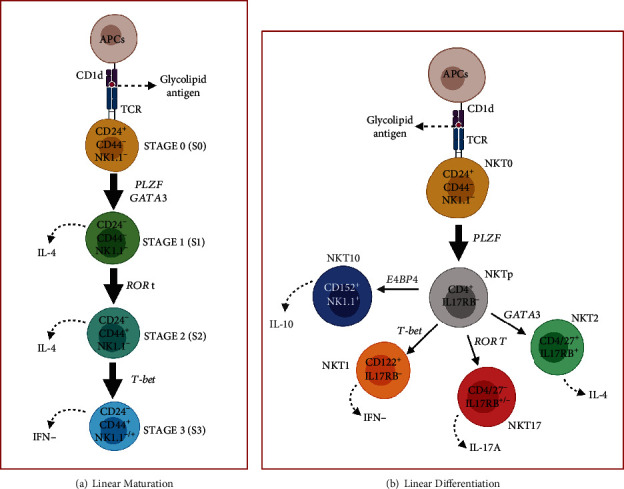
Maturation and differentiation of NKT cells. (a) According to the linear maturation model, mice NKT cells go through four maturation stages, from S0 to S3. After antigen presentation, NKT cells progress from an immature stage S0 as CD24^+^CD44^−^NK1.1^−^ cells to stage S1 as IL-4-producing CD24^−^CD44^−^NK1.1^−^ cells, depending on the activation of transcription factors PLZF and GATA3. After activation of the transcription factor ROR*γ*t, cells mature to stage S2 as CD24^−^CD44^+^NK1.1^−^ cells, which also produce IL-4. Finally, activation of the transcription factor T-bet promotes maturation to state S3 as CD24^−^CD44^+^NK1.1^-/+^ cells, which produce IFN-*γ*. (b) After the antigenic presentation, NKT0 EGR2^+^CD24^+^CD44^−^NK1.1^−^ progenitor cells differentiate to CD4^+^IL17RB^−^ precursor cells in a manner dependent on the activation of the transcription factor PLZF. After the activation of the transcription factors T-bet, GATA3, ROR*γ*t, or E4BP4, the precursor NKT cells acquire the potential to differentiate into IFN-*γ*-producing NKT1 T-bet^+^CD122^+^IL17RB^−^, IL-4-producing NKT2 GATA3^+^CD4^+^CD27^+^IL17RB^+^, IL-17A-producing NKT17 ROR*γ*t^+^CD4^−^CD27^−^IL17RB^+/-^, or IL-10-producing NKT10 E4BP4^+^CD152^+^NK1.1^+^ cells. APCs, antigen-presenting cells; CD, cluster of differentiation; EGR2, early growth response protein-2; E4BP4, transcription factor E4 promoter-binding protein 4; GATA3, transcription factor GATA binding protein 3; IFN, interferon; IL, interleukin; NK1.1, Natural Killer cell pectin-like receptor subfamily B molecule member 1; NKT, Natural Killer T cell; NKTp, Natural Killer T cell precursor; NKT0, Natural Killer T cell progenitor; PLZF, promyelocytic leukemia zinc finger; ROR*γ*t, transcription factor retinoic acid-related orphan nuclear receptor *γ*t; T-bet, transcription factor T-box; TCR, T-cell receptor. (Created with http://biorender.com).

Until acquiring their final functional phenotype, mice NKT cells go through four well-defined maturation stages (stages S0 to S3), characterized by the expression of specific surface markers, a process called linear maturation [19, 20]. On the basis of this linear maturation model, NKT cells progress from an immature stage S0 as CD24^+^CD44^−^NK1.1^−^ cells, through stages S1 and S2 as CD24^−^CD44^−^NK1.1^−^ and CD24^−^CD44^+^NK1.1^−^ cells, respectively, to reach the final maturation phenotype during the stage S3 as CD24^−^CD44^+^NK1.1^-/+^ cells [19, 20] (Figure 2(a)).

In addition, mice NKT cells have been divided into three functional subsets, termed NKT1, NKT2, and NKT17 cells, based on the transcription factors and surface markers they express and the cytokines they produce [19, 20]. In this context, the progenitor NKT0 CD24^+^CD44^−^NK1.1^−^ cells express the EGR2 transcription factor and, after antigenic recognition and activation of the PLZF transcription factor, they differentiate into NKT1, NKT2, or NKT17 cells [9, 20–22] (Figure 2(b)). Once activated, NKT1 cells present the phenotype markers PLZF^low^Tbet^+^CD122^+^IL17RB^−^ and produce IFN-*γ* and low levels of IL-4, being the only NKT subset that expresses proteins characteristic of NK cells: NK1.1, NKG2D, and Nkp46 [9, 20–22]. Otherwise, NKT2 cells have the phenotype PLZF^high^GATA3^+^CD4^+^CD27^+^IL17RB^+^ and produce increased levels of IL-4, and NKT17 cells have the phenotype PLZF^me^ROR*γ*T^+^CD4^−^CD27^−^IL17RB^+/-^ and produce high levels of IL-17A [9, 20–22]. Recently, it was established that the activation of the Hivep3 transcription factor in early precursors regulates the post-selection proliferative burst and differentiation of NKT cells and that the upregulation of Fcɛr1*γ* and SerpinB1 are involved in the selective generation of NKT1 and NKT17cells, respectively [23, 24]. According to the linear differentiation model, only NKT1 cells go through all stages, S0 to S3, of the linear maturation model, unlike NKT2 and NKT17 cells that acquire their functional maturation in stage S2 [20]. In summary, IFN-*γ*-producing NKT1 cells, IL-4-producing NKT2 cells, and IL-17A-producing NKT17 cells are considered terminally differentiated cells, lacking the ability to transdifferentiate into another effector NKT phenotype [22, 25].

However, in the case of humans, the differentiation of the NKT subsets has not been fully described. In peripheral blood, two subpopulations of NKT cells have been described, the V*α*24 CD4^−^CD8^−^ and V*α*24 CD4^+^ NKT cells, which represent functionally different lineages that express different pattern of cytokines, chemokine receptors, and membrane integrins [26]. In particular, CD4^−^CD8^−^ NKT cells produce high levels of IFN-*γ* and express surface markers of the NK lineage, while CD4^+^ NKT cells produce high levels of IL-4 and IL-13, in a similar way to NK1 and NK2 cell subsets, respectively [26].

## 3. Nature of the Glycolipids Binding to CD1d

The glycolipids binding to CD1d are oligosaccharide structures bound to lipidic residues, which can have a microbial origin (natural glycolipids) or can be artificially generated (synthetic glycolipids) to experimentally induce its development and immune activity [27–29]. In bacteria, the molecular nature of immunogenic glycolipids is diverse and includes *α*-galactosyldiacylglycerol, expressed by *Borrelia burgdorferi*, lipophosphoglycan, expressed *Leishmania donovani*, *α*-glucosyldiacylglycerol, expressed by *Streptococcus pneumoniae*, *α*-glucuronosylceramide and *α*-galacturonosylceramide, expressed by *Sphingomonas* species, and *α*-glucoside, expressed by *Helicobacter pylori* [30–34]. When these glycolipids interact with the APCs, their cellular internalization is dependent on the recognition by the low-intensity lipoprotein receptor (LDLR) as well as their interaction with the scavenger receptor (SR) type A (SRA), SRB1, and CD36, leading to the CD1d-glycolipid conjugation and the activation of the NF-*κ*B and NF-AT signaling pathways, required for the subsequent induction of NKT cells [35, 36]. During the glucolipid presentation to the NKT cells, the coupling of the CD1d-glycolipid complex with the TCR results of the high-affinity interaction between the *α*-chain of the TCR with the sugar ring of the glycolipid and the stabilizing interaction of the *β*-chain of the TCR with CD1d, triggering the NKT cell activation [37].

In an experimental manner, human and mice-derived NKT cells can also be activated with a synthetic glycolipid called *α*-galactosylceramide (*α*-GalCer) [11, 38]. In this case, the CDR1*α* and CDR3*α* complementarity regions of the TCR interact with *α*-GalCer, while CDR2*β* interacts with CD1d [39]. The *α*-GalCer molecule derives from the marine sponge *Agelas mauritanius* and is defined by the presence of anomeric glycosidic bonds with galactose residues attached to the base of sphingosine [11, 38]. Moreover, for experimental purposes, an analog of *α*-GalCer was recently developed, known as KRN7000 [11]. When human or murine NKT cells are stimulated with *α*-GalCer or KRN7000, they activate very efficiently due to their high affinity to CD1d [40, 41]. Indeed, they are capable of activating both progenitor NKT cells and mature NKT cells, leading to the production of high levels of cytokines [38, 42, 43].

## 4. Anatomical Distribution of NKT Cells

**Figure 3:**
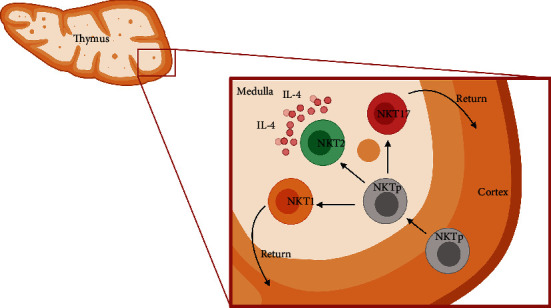
Distribution of NKT cells during their maturation in the mice thymus. During their maturation process, the NKT precursor cells located in the thymic cortex migrate towards the medulla to maturate and acquire the phenotypes NKT1, NKT2, or NKT17. In the thymic medulla, steady-state NKT2 cells produce IL-4 and promote the activation of medullar thymocytes. Conversely, NKT1 and NKT17 cells return to the cortical area. IL, interleukin; NKT, natural killer T cell; NKTp, Natural Killer T cell precursor. (Created with http://biorender.com).

Effector NKT1, NKT2, and NKT17 cells are distributed differently among the distinct lymphoid organs and have particular migratory patterns associated with their maturation [19]. In the mice's thymus, progenitor NKT0 cells are located in the thymic cortex [8, 19]. While 70% of NKT1 and NK17 cells reside in the thymic medulla and 30% in the cortex, 90% of NKT2 cells are located in the medulla [8, 19, 44]. During the maturation process, NKT0 cells migrate from the thymic cortex towards the medulla to acquire the phenotype NKT1, NKT2, or NKT17; otherwise, NKT1 and NKT17 cells return to the cortex, and NKT2 cells remain in the thymic medulla after maturing [16, 19] (Figure 3). Currently, the cause and mechanism involved in the return of NKT1 and NKT17 cells from the medulla towards the thymic cortex have not been clearly defined, neither why NKT2 cells do not perform this migration and remain in the thymic medulla [16, 19, 44].

The differential distribution of the NKT cell subsets in the thymus and their migration towards the peripheral tissues seems to be dependent on the chemokine receptors that they express. Indeed, the retention of mature NKT cells in the thymic medulla is associated with the surface expression of the chemokine receptor CXCR3, which interacts with its specific ligand CXCL10, expressed by medullary thymic epithelial cells (mTECs) [45, 46]. After activation and under inflammatory conditions, the IFN-*γ* production by mature CXCR3^+^ NKT cells seems to downregulate the CXCL10 expression by mTECs, which would release the CXCR3-CXCL10-linked NKT-mTEC interaction and lead to the increased production of CXCL9, CXCL10, and CXCL11 in compartments such as the endothelium, favoring their migration towards peripheral tissues [45, 46].

Besides, mTECs express lymphocytoxin-*β* receptor (LT*β*R), which is essential in the regulation of the mTEC heterogeneity as an intrathymic mechanism that determines the development and function of NTK cells and their selective migration towards peripheral tissues [47]. Three distinct mTEC subsets have been described, which enable the thymus to differentially control NKT sublineages [47]. In this context, mTEC expression of LT*β*R controls the development of thymic tuft cells, which determines the development of NKT2 cells via IL-25, while LT*β*R controls CD104^+^CCL21^+^ mTECs that are capable of IL-15-transpresentation for the determination of the NKT1 and NKT17 cell development [47].

The CD28 co-stimulation has also been involved in the maturation, activation, and proliferation processes of thymus resident NKT cells [48]. In fact, blocking CD28 signaling in stage S3 NKT cells diminishes their proliferative potential in comparison with stage S2 NKT cells [48]. Importantly, PLZF is upregulated in undivided NKT cells despite the blockade of CD28, suggesting that PLZF expression is TCR-dependent and CD28-independent [48, 49]. Thus, TCR signaling induces PLZF upregulation in a process prior to cell proliferation, and the absence of CD28 signaling maintains NKT cells in a non-dividing state, which causes an increased PLZF expression when CD28 is blocked [48, 49].

In the mice spleen, NKT1 cells are located in the red pulp and NKT2 cells are located in the white pulp [8, 19]. In mice lymph nodes, NKT1 cells are mainly located in the distribution zones of both T lymphocytes and B lymphocytes, while NKT2 cells are preferentially located next to the T lymphocytes [16, 19, 44]. Similarly, NKT17 cells are located in the sub-capsular area in a comparable way to Th17 lymphocytes [21]. Recently, it has been established that the liver contains a high number of NKT1 cells and a low number of NKT2 and NKT17 cells [22], which has been ratified experimentally after the administration of *α*-GalCer [19, 22]. Indeed, intravenous administration of *α*-GalCer induced a high production of IL-4 and IFN-*γ* in NKT1 cells located in the splenic red pulp [19, 22]. Conversely, oral administration of *α*-GalCer induced the production of IL-4 but not IFN-*γ* in NKT2 cells located in mesenteric lymph nodes, which favored the paracrine activation of infiltrating T lymphocytes without systemic effects [19, 44]. Thus, the topographic tissue distribution of mice NKT effector cells in different immune organs could be associated with their functional potential, particularly with the local or systemic production of IL-4 and IFN-*γ* [19, 44]. In contrast, the distribution of NKT effector cell subsets in humans has not been described in detail yet.

## 5. NKT Cells and Activation of T Lymphocytes

**Figure 4:**
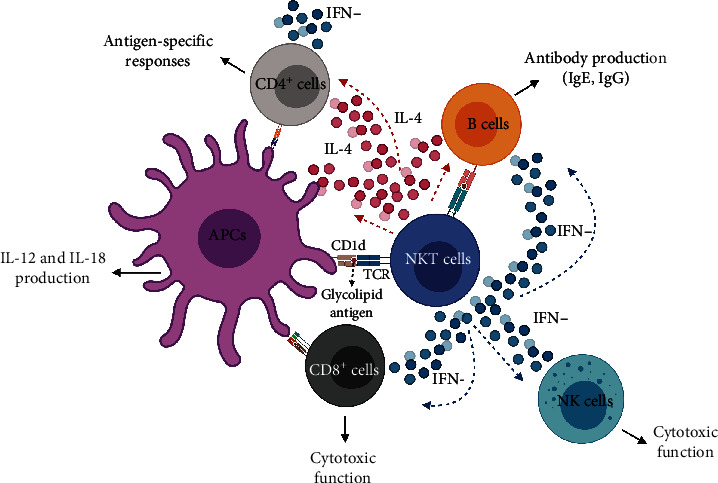
NKT cell-mediated transactivation. Following antigen presentation, NKT cells rapidly produce high levels of IFN-*γ* or IL-4. IFN-*γ*-producing NKT1 cells promote the lytic activity of CD8^+^ lymphocytes and NK cells and the production of IgE and IgG in B lymphocytes. On the other hand, IL-4-producing NKT2 cells promote the production of IL-12 and IL-18 in APCs, the production of IgE and IgG in B lymphocytes, and the production of IFN-*γ* and capacity for specific antigen recognition in CD4^+^ T lymphocytes. APCs, antigen-presenting cells; CD, cluster of differentiation; IFN, interferon; Ig, immunoglobulin; IL, interleukin; NK, Natural Killer cell; NKT, Natural Killer T cell; TCR, T-cell receptor. (Created with http://biorender.com).

Murine and human effector NKT cells produce high levels of cytokines immediately after being stimulated [11, 50]. This ability to rapidly produce cytokines favors the activation of other immune cell types, such as antigen-presenting cells, NK cells, B lymphocytes, CD4^+^ helper T lymphocytes, and CD8^+^ cytotoxic T lymphocytes, a process termed transactivation [11, 50] (Figure 4).

The transactivation of T helper lymphocytes depends on the solubility of the antigenic glycolipid that stimulates the NKT cells [11, 50]. Structural changes that increase the hydrophobicity of antigenic glycolipids favor the selective polarization of T helper lymphocytes towards the Th1 phenotype, and thus, a proinflammatory response [11]. In contrast, antigenic glycolipids with greater solubility in aqueous environments favor the differentiation and anti-inflammatory activity of Th2 lymphocytes [11]. In this context, the induction of a Th1 lymphocyte response is established when APCs internalize the antigenic glycolipid, to be conjugated to a CD1d molecule, within endosomal compartments in a hydrophobic environment and a low pH-dependent manner [11, 51]. In this way, the glycolipid/CD1d complex presentation is linked to membrane microdomains of endosomal origin, called lipid rafts [11]. In contrast, hydrophilic glycolipids promote a Th2-type anti-inflammatory response by directly associating with CD1d molecules in an endosome-independent manner [11]. Together, it is proposed that the solubility of the antigenic glycolipids determines the mechanism of interaction with the CD1d molecule and, thus, the activation of NKT cells and Th1/Th2 transactivation [11, 52].

In this sense, the activation of NKT1 cells with hydrophobic glycolipids would favor the rapid production of IFN-*γ* and the consequent transactivation of Th1 lymphocytes, while the activation of NKT2 cells with hydrophilic glycolipids would favor the rapid production of IL-4 and the consequent transactivation of Th2 lymphocytes [7, 11, 51, 52]. In this context, the particular condition dependent on the solubility of antigenic glycolipids that determine the activation of NKT17 cells and potential transactivation of Th17 lymphocytes has not been studied in detail.

The functional association between NKT and T-cell subsets could also be transferred to the innate lymphoid cells (ILCs), since the transcription factors and cytokines expressed by the distinct ILC subsets are also overlapping with those expressed by NKT1, NKT2, and NKT17 cells [53]. Indeed, ILC1s express the transcription factor T-bet and produce high levels of IFN-*γ*, similar to NKT1 cells, ILC2 cells express GATA-3 and produce IL-4 and IL-5, similarly to NKT2 cells, and ILC3 cells express ROR*γ*T and produce high levels of IL-17A and IL-22, similar to NKT17 cells [53–55]. Thus, ILCs and NKT cells could play a fundamental collaborative role in the maintenance of immune homeostasis and could promote a phenotype-specific NKT/T-cell/ILC transactivation in different disease conditions [53, 54]. In this context, the NKT1/Th1/ILC1, NKT2/Th2/ILC2, and NKT17/Th17/ILC3 responses could be coordinately deployed and together be responsible for the pathogenic changes in some diseases.

## 6. Pathologic Role of NKT1, NKT2, and NKT17 Cells

NKT cells have been associated with the pathogenesis of several diseases with diverse etiologies. In these pathological conditions, both pathological and protective NKT activities have been reported, depending on the type of pathology, its severity, the involved NKT subset, the time of exposure to antigens, and the methodology used to analyze the NKT activity [14, 56, 57].

### 6.1. Metabolic Diseases

NKT cells have been detected in high numbers in the liver and adipose tissue in mice, and due to their glycolipid reactivity, NKT cells have been associated with the development of non-alcoholic fatty liver disease (NAFLD), obesity-associated inflammation, and resistance to insulin [58]. In the pathogenesis of NAFLD, hepatic NKT cells have shown a dual role, being protective during the early phase of the disease while promoting pathological fibrogenesis in later stages of more severe NAFLD [59, 60]. In murine models of obesity fed with a diet high in fatty acids, hepatic and adipose NKT cells are rapidly activated and, consequently, promote tissue inflammation, insulin resistance, and hepatic steatosis [61]. On the other hand, the adoptive transfer of NKT cells into leptin-deficient murine leads to the reduction of fatty liver and glycemia [62]. Similarly, the adoptive transfer of NKT cells into murine fed with a low-fatty acid diet promotes insulin resistance, which does not occur when NKT cells are transferred into animals fed with a diet high in fat [63]. Indeed, in CD1d and J*α*18-null mice fed with a diet low in fat, a decrease in insulin resistance was observed [64].

### 6.2. Autoimmune Diseases

During murine autoimmune encephalomyelitis, the IFN-*γ*-producing NKT1 cells favor the pathogenic response of Th1 and Th17 lymphocytes in the central nervous system [65]. Conversely, IL-4-producing NKT2 cells inhibit the Th1 response, but not the pathogenic Th17 response [66, 67]. In a murine model of alopecia areata induced by scalp xenografts and inoculation of PBMCs stimulated *in vitro* with IL-2, an increase in the number of NKT1 cells was observed [68]. In turn, this increase in the NKT1 cell number led to the expansion of T helper and cytotoxic lymphocytes and an increment in the production of IFN-*γ*, causing hair follicle dystrophy and focal hair loss [68]. In different mice models of systemic lupus erythematosus, NKT cells have been associated with an increase in lupus nephritis and the appearance of severe skin lesions [14, 69, 70]. In SJL/J mice in which lupus was induced by inoculation of pristane (tetramethyl-pentadecane), the administration of *α*-GalCer favored the expansion and activation of NKT cells with proinflammatory activity [69]. Indeed, the long-term administration of an IgG2a anti-NK1.1 monoclonal antibody ameliorated the lupus-like disease in NZB/W mice, demonstrating the pathological role of NKT cells [71]. In autoimmune hepatitis, an increased number of TNF-*α*-producing NKT cells was observed in the liver and peripheral blood, which were characterized by producing reduced levels of IFN-*γ* and the absence of IL-4 [72]. In this way, they contributed to exacerbate the pathological inflammation.

### 6.3. Bone Resorptive Diseases

During rheumatoid arthritis, NKT cells play a pathological role by promoting the progressive destruction of articular bone [14, 70]. In rheumatoid arthritis-affected patients, abundant infiltration of NKT1 cells has been observed in the affected joints [15]. Indeed, in an animal model of collagen-induced rheumatoid arthritis (CIA), long-term administration of *α*-GalCer induced the activation of infiltrating NKT cells, which promoted the differentiation of T helper lymphocytes with proinflammatory activity [14]. Otherwise, in osteoporosis-affected patients, bone damage has been directly associated with the activity of NKT1 and NKT17 cells [17]. Indeed, NKT cells favor the increase of receptor activator of nuclear factor *κΒ* ligand (RANKL) expression levels, the key factor responsible for osteoclastogenesis and osteoclast-mediated bone resorption, a phenomenon that rises with the severity of the disease and depends on the loss of estrogens [17].

## 7. Pathologic Role of NKT1, NKT2, and NKT17 Cells during Periodontitis

Periodontitis results from polymicrobial synergy and dysbiosis of the bacterial communities colonizing the subgingival environment; however, periodontal tissue breakdown is mainly determined by the host's immune response, where the T-lymphocyte subpopulations play a key role in the alveolar bone resorption that leads to tooth loss [73, 74]. Indeed, alveolar bone resorption involves the antagonistic relationship between the activity of RANKL-producing Th17 lymphocytes and IL-10-producing T regulatory (Treg) lymphocytes [75, 76]. Thus, alveolar bone resorption is, at least in part, a consequence of the Th17/Treg imbalance of the immune response deployed in the periodontitis-affected tissues, which causes the upregulation of RANKL, and other proinflammatory cytokines, that further promote osteoclastogenesis and alveolar bone loss [77–79].

Various *in vivo* and *in vitro* studies have established the pathogenic role of NKT cells during periodontitis [80]. In this context, Gram-negative bacteria contain glycosphingolipids in their outer membrane; thus, periodontal pathogenic bacteria, such as *Porphyromonas gingivalis*, *Aggregatibacter actinomycetemcomitans*, *Tannerella forsythia*, and *Treponema denticola* may express glycosphingolipids in their structure, which could be processed and presented by CD1d^+^ APCs and consequently activate NKT cells [81–83]. After oral inoculation of *P*. *gingivalis* and intraperitoneal administration of *α*-GalCer in mice, NKT cells promoted systemic inflammation, periodontal RANKL production, osteoclastogenesis, and alveolar bone loss [84, 85]. Interestingly, when periodontitis was induced in CD1d^−/−^ mice, oral inoculation of *P. gingivalis* triggered lesser alveolar bone resorption as compared with periodontitis induced in wild-type mice [84, 85]. When human-derived NKT cells were exposed *in vitro* to dendritic cells stimulated with *A. actinomycetemcomitans*, they secreted higher levels of IFN-*α*, IFN-*β*, and IFN-*γ*, in a dose-dependent manner, in comparison with NKT cells exposed to *P. gingivalis*-primed dendritic cells [86, 87]. These results detected in NKT cells confirm the greater immunogenic capacity of *A. actinomycetemcomitans* over *P. gingivalis* also described in other immune cells [88–90].

In periodontitis-affected tissues, T lymphocytes with invariant TCR V*α*24/J*α*Q have been detected as the dominant clone among V*α*24^+^ cells, and this cell population includes NKT cells [91]. In gingival biopsies obtained from periodontitis or gingivitis-affected patients, the expression of the marker V*α*24 and the different isoforms of CD1 were analyzed, observing a predominance of CD1d over CD1a, CD1b, and CD1c isoforms and higher number of CD1d^+^ cells and V*α*24^+^ NKT cells in periodontitis patients, as compared with gingivitis patients [83]. Although dendritic cells can express CD1d, the analysis of CD1d and CD83 double expression revealed a significant number of infiltrating CD1d^+^CD83^−^ cells in periodontal lesions, which do not correspond to dendritic cells [83]. This indicates that other APCs that share the CD1d molecule could be involved in the antigenic presentation and activation of NKT cells during periodontitis [42, 83]. Indeed, the co-expression of CD1d and CD19 on the same cells and proximate infiltration of CD1d^+^ and V*α*24^+^ cells was observed in periodontitis-affected tissues, suggesting that CD1d^+^ B lymphocytes could activate V*α*24^+^ NKT cells in a CD1d-restricted manner [83], being B lymphocytes largely present in the diseased gingival tissues infiltrate of periodontitis-affected patients [92]. Moreover, human NKT cells have the capacity to recognize antigens presented by CD1d on B lymphocytes, and in turn, activated NKT cells can induce B-cell proliferation and immunoglobulin production [83, 93]. Thus, the pathogenic role of NKT cells during periodontitis could be tightly linked to the activity of B lymphocytes, both in their role as CD1d-restricted APCs and as antibody-producing effector cells.

Consistent evidence has demonstrated the central role of RANKL in bone homeostasis and pathological bone resorption [76]. Even so, RANKL has also been associated with other physiological processes, such as alveologenesis during the pregnancy-associated mammary gland development and thymic selection of immune cells to acquire the property of distinguishing self-antigens from non-self-antigens [94–96]. During thymic selection, NKT cells express RANKL in their membrane, which in turn interacts with the RANK receptor expressed in mTECs and leads to the activation of the transcription factor AIRE, the expression of MHC associated with tissue-specific antigens, and the promotion of apoptosis of the recipient cells [96–98]. To date, the presence of NKT cells that produce RANKL in the peripheral tissues has not been demonstrated; however, their existence would allow to hypothesize that RANKL-producing NKT cells could be associated with the immune control of bone loss during osteolytic diseases, including periodontitis.

**Figure 5:**
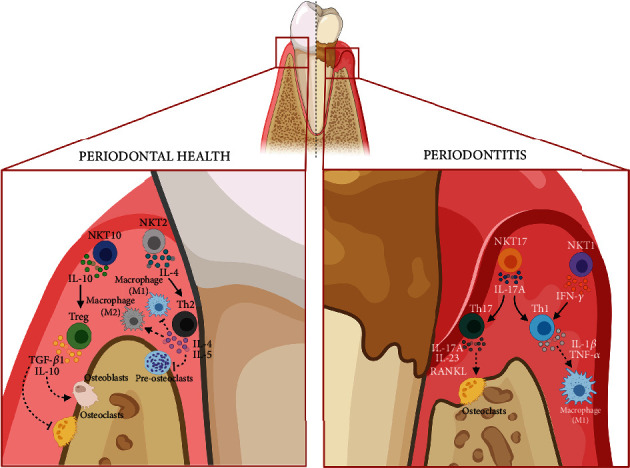
Role of NKT cells during periodontal health and disease. During periodontal health, the low-grade antigenic load of commensal microbial origin favors the differentiation and activity of NKT2 cells and eventually NKT10 cells. In this context, NKT2 and NKT10 cells could promote the transactivation of Th2 and Treg lymphocytes, which inhibit osteoclastogenesis and M1 macrophage activation, and induce osteoblastogenesis and M2 macrophage activation, thus favoring periodontal tissue homeostasis. During periodontitis, the host's immune response is triggered against the dysbiotic bacterial communities colonizing the subgingival environment. During this process, the antigen-presenting cells present the microbial and tissue-damage antigens to the distinct immune effector cells; therefore, they can present antigens of a glycolipid nature to NKT cells. In this context, activated NKT cells acquire the potential to differentiate into NKT1 and NKT17 cells. Then, NKT1 and NKT17 cells could promote the transactivation of Th1 and Th17 lymphocytes, which induce the activation of M1 macrophages and the differentiation and function of osteoclasts, and consequently, promote periodontal inflammation and alveolar bone resorption. IFN, interferon; IL, interleukin; Treg, regulatory T lymphocytes; NKT, Natural Killer T cell; RANKL, receptor activator of nuclear factor *κΒ* ligand; TGF, transforming growth factor; TNF, necrosis factor tumors. (Created with http://biorender.com).

Although these antecedents could allow us to establish a clear association between the activity of NKT cells and the pathological events that characterize periodontitis, to date, there is no clear evidence that supports the direct relationship of a particular NKT subset with the pathogenesis of the disease. The currently accepted paradigm that contributes to explain the pathogenesis of periodontitis involves an immuno-inflammatory response mediated by the activity of Th1, Th2, Th17, and Treg lymphocytes, where Th1 and Th17 lymphocytes have been more related to the onset and progression of periodontitis, while Th2 and Treg lymphocytes have been more related to the suppression of the Th1 and Th17 responses, periodontitis remission, and periodontal healing [86, 99–105]. However, a functional relationship between Th1, Th2, or Th17 lymphocytes and NKT1, NKT2, or NKT17 cells during periodontitis has not been established yet. Given the similarity of their cytokine profile that justifies their names, it is plausible to hypothesize a functional relationship between the Th1/NKT1, Th2/NKT2, and Th17/NKT17 cells (Figure 5). In this sense, a periodontal protective relationship could also be observed between Treg lymphocytes and the recently described NKT10 cells, which produce IL-10 and fulfill regulatory activities [66, 67, 106, 107].

## 8. NKT10 Cells: A New NKT Subset with Regulatory Functions

Recently, a new subset of NKT cells with regulatory capacity characterized by their IL-10 production has been described, the reason they are termed NKT10 cells [5, 108]. The functional capacities of NKT10 are the subject of current research; even so, it has been clearly established that, after glycolipid antigenic stimulation, NKT10 cells reduce their capacity to produce proinflammatory cytokines and largely increase their IL-10 production [5, 108]. In this way, NKT10 cells are able to favor and enhance immunoregulatory responses by promoting the clonal expansion and activity of Treg lymphocytes and M2 macrophages [5, 108, 109]. Indeed, NKT10 cells express several common markers with Treg lymphocytes, including the surface markers CD152 (CTLA4), CD279 (PD1), and CD304 (Neuropilin-1) [5, 108]. In mice, NKT10 cells are located mainly in the thymus, spleen, and adipose tissue; in the latter, NKT10 cells correspond to ~15% of the activated NKT cells [108, 110], and in humans, 0.5% of the circulating blood cells are NKT10 cells [5, 108].

The regulatory potential attributed to IL-10-producing NKT10 cells has led to emerging proposals targeting them as a novel therapy for various inflammatory diseases, in such a way that NKT10 activation and expansion could favor a protective immune response [5, 108]. For instance, during autoimmune encephalomyelitis in mice, NKT10 cells inhibit the pathogenic activity of Th1 lymphocytes in the central nervous system [66, 67]. Similarly, in a murine model of alopecia areata, inoculation of IL-2-pulsed PBMCs previously exposed to NKT10 cells led to the inhibition of the generation of alopecic lesions, demonstrating the protective role of NKT10 cells in this disease [107]. In the same way, in an animal model of systemic lupus erythematosus, short-term stimulation with *α*-GalCer induced an increase in IL-10 production, which led to less secretion of autoantibodies and an increase in the production of tolerogenic cytokines [106]. Thus, NKT10 cells could fulfill a protective role in the pathogenesis of lupus erythematosus, related to their ability to regulate the production of autoreactive IgG by CD1d^+^ B lymphocytes [106].

The inoculation of *α*-GalCer can promote the expansion of NKT10 cells and an anti-inflammatory environment mediated by the production of Th2 and Treg-related cytokines in diverse diseases [66, 108, 111]. In this sense, the activation of NKT10 cells favors the IL-10 mediated transactivation of Treg lymphocytes, and thus, the regulation of pathological responses [108]. To date, the regulatory role of NKT10 cells and their possible functional relationship with Treg lymphocytes during periodontitis has not been demonstrated yet, making the immuno-therapeutic proposal based on the induction of NKT10 cells by stimulation with *α*-GalCer or other glycolipid antigens to control periodontitis, in our opinion, attractive. In this way, the induction of NKT10 activity in infected periodontal tissues could lead to the transactivation of Treg lymphocytes, the promotion of phenotypic stability of Treg lymphocytes, and the recovery of the Th17/Treg balance.

## 9. Concluding Remarks

Multiple lines of evidence point to the pathogenic role of NKT cells in diverse inflammatory and osteolytic diseases. Similarly, consistent evidence has established that an increase in effector NKT cell activity promotes systemic inflammation, local RANKL production, and alveolar bone resorption during periodontitis. To date, there is no clear evidence that describes the pathogenic role of a specific NKT subset during periodontitis; however, the ability of NKT1 and NKT17 cells to produce proinflammatory cytokines, specifically IFN-*γ* and IL-17A, respectively, broadens the conceptual paradigm that allows us to understand the pathogenesis of periodontitis. In this context, the recent discovery of NKT10 cells with regulatory potential allows us the possibility of exploring a new therapeutic target with the potential to regulate the Th17/Treg imbalance that characterizes the disease. Thus, further studies are required to unravel the pathogenic role of NKT1 and NKT17 cells and the regulatory role of NKT10 cells during periodontitis.

## Data Availability

No data were used to support this study.
